# Activating the Microscale Edge Effect in a Hierarchical Surface for Frosting Suppression and Defrosting Promotion

**DOI:** 10.1038/srep02515

**Published:** 2013-08-28

**Authors:** Xuemei Chen, Ruiyuan Ma, Hongbo Zhou, Xiaofeng Zhou, Lufeng Che, Shuhuai Yao, Zuankai Wang

**Affiliations:** 1 Department of Mechanical and Biomedical Engineering, City University of Hong Kong, Hong Kong 999077, China; 2 Department of Mechanical Engineering, The Hong Kong University of Science and Technology, Hong Kong 999077, China; 3 State Key Laboratory of Transducer Technology, Shanghai Institute of Microsystem and Information Technology, Chinese Academy of Sciences, 865 Changning Road, Shanghai 200050, China; 4 Shenzhen Research Institute of City University of Hong Kong, Shenzhen, China

## Abstract

Despite extensive progress, current icephobic materials are limited by the breakdown of their icephobicity in the condensation frosting environment. In particular, the frost formation over the entire surface is inevitable as a result of undesired inter-droplet freezing wave propagation initiated by the sample edges. Moreover, the frost formation directly results in an increased frost adhesion, posing severe challenges for the subsequent defrosting process. Here, we report a hierarchical surface which allows for interdroplet freezing wave propagation suppression and efficient frost removal. The enhanced performances are mainly owing to the activation of the microscale edge effect in the hierarchical surface, which increases the energy barrier for ice bridging as well as engendering the liquid lubrication during the defrosting process. We believe the concept of harnessing the surface morphology to achieve superior performances in two opposite phase transition processes might shed new light on the development of novel materials for various applications.

Engineering “icephobic” surfaces that can retard the frost formation and accumulation is of scientific and practical importance. Frost formation and accumulation on cold surfaces adversely affect the operational performance in aircrafts, refrigerators, wind turbines and power lines^1^,^2^,^3^,^4^,^5^. Current approaches to developing durable icephobic surfaces focus on two research lines. One is the development of roughness-induced superhydrophobic surfaces with small contact angle hysteresis^6^,^7^,^8^,^9^,^10^,^11^,^12^,^13^,^14^,^15^,^16^,^17^,^18^,^19^ and the other is based on lubricant-infused surfaces^20^,^21^,^22^. In the research line of roughness-induced superhydrophobic surfaces, the studies of frost formation mainly focus on individual droplets, either deposited^6^,^7^,^8^,^9^,^10^,^11^ or impacted^12^,^13^,^14^,^15^,^16^. However, the working conditions encountered in industrial applications are more conducive to condensation frosting (formation of supercooled condensate and subsequent freezing into frost)^23^,^24^,^25^,^26^. For the condensation frosting process, the frost formation is inevitable owing to the inter-droplet freezing wave propagation across the entire surface initiated from the surface edges or defects, where heterogeneous ice nucleation is more favored^27^,^28^,^29^. On the other hand, without a delicate control of surface morphology and chemistry, the frost adhesion on the micro/nanostructured superhydrophobic surface is increased due to its significantly enlarged total surface area^30^,^31^,^32^,^33^,^34^,^35^,^36^,^37^,^38^, which in turn compromises the icephobic properties of the superhydrophobic surface and increases the operation cost and energy consumption in the defrosting process. Here, by exploiting the controlled microscale edge effect and synergistic cooperation of two-tier roughness, we report a hierarchical micro/nanostructured superhydrophobic surface that not only significantly suppresses the ice nucleation and inter-droplet freezing wave propagation in the condensation frosting process, but also promotes fast frost removal in the defrosting stage.

## Results

### Inter-droplet freezing wave propagation dynamics

**Figure 1:**
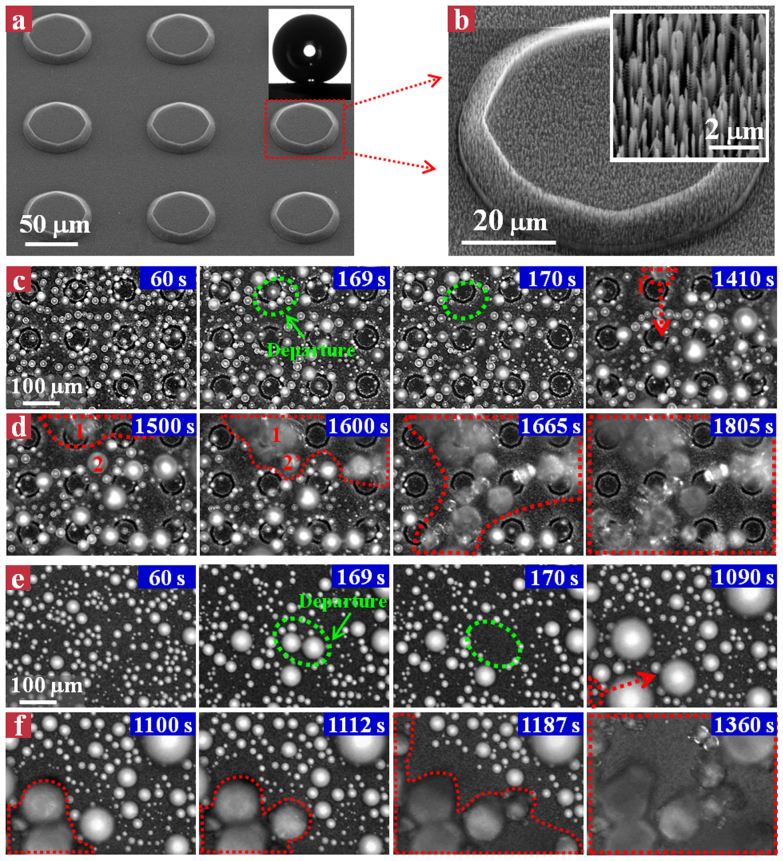
Condensation frosting processes on the hierarchical and nanograssed superhydrophobic surfaces. (a) Scanning electron microscopy (SEM) image of hierarchical surface with nanograssed micro-truncated cone architecture. The inset shows a 4 μL water droplet deposited on the hierarchical surface with a contact angle of ~166°. (b) A close-up SEM image showing an individual nanograssed micro-truncated cone. The inset shows the SEM image of the nanograss arrays that are coated on the micro-truncated cone. (c) Droplet freezing dynamics on the hierarchical surface at −10°C. Initially, the hierarchical surface stays in a dropwise condensation stage, with small spherical condensate droplets growing over time and constantly departing from the surface (as revealed by the green dashed line). Condensate droplets maintain their liquid state until a freezing wave invading the field-of-view, as evidenced by the rapid onset of opacity in droplet 1 circled by the red line at time of 1410 s. (d) Inter-droplet freezing wave propagation on the hierarchical surface. As the time proceeds, the inter-droplet freezing wave gradually propagates across the entire field-of-view within 395 s. (e) Droplet freezing dynamics on the nanograssed surface at −10°C. The condensate droplets exhibit remarkable jumping, though relatively less frequent compared to those on the hierarchical surface. Condensate droplets maintain their liquid state until a freezing wave invading the field-of-view at ~1090 s. (f) Inter-droplet freezing wave propagation on the nanograssed surface. As the time progresses, the inter-droplet freezing wave gradually propagates across the entire field-of-view within ~260 s, which is 50% faster than that on the hierarchical surface.

We first designed and fabricated a hierarchical surface with nanograssed micro-truncated cone architecture (see Methods Section for details). The hierarchical surface was fabricated with a two-step process we developed previously^39^,^40^. Briefly, the micro-truncated cone structure with an inclination angle of 54.7° was first created using an anisotropic wet-etching, and then nanograss arrays were etched on the whole surface using a modified deep reactive ion etching (DRIE) process^41^,^42^,^43^,^44^,^45^. The top and base diameters of the truncated cones are ~55 and ~70 μm, respectively. The pitch between truncated cones is ~50 μm and the height of the truncated cones is ~10 μm (Fig. 1a–b). The nanograsses are ~300 nm in diameter, ~5 μm in height, and ~200–350 nm in pitch (as shown in the inset of Fig. 1b). The as-fabricated surface was silanized in the hexane solution of perfluorooctyl trichlorosilane for 30 min, followed by heat treatment at 150 °C for 1 h. After surface modification, the hierarchical surface exhibits a contact angle of ~166° and contact angle hysteresis less than 1°. The condensation frosting experiment was carried out in an environment with an ambient temperature of 22 °C and relative humidity (RH) of 65%. In order to avoid the gravitational effect, we put the sample (9 mm × 9 mm) horizontally on the cooling stage with a preset temperature of −10 °C.

We systematically studied the time evolution of condensation frosting dynamics on the as-developed hierarchical superhydrophobic surface. We found that droplet freezing primarily begins from the outer edge corners of the substrate owing to its geometric singularity and low free energy barrier for heterogeneous nucleation. This edge effect triggers the formation of inter-droplet freezing wave which propagates across the entire surface^27^,^28^,^29^. In order to evaluate the anti-freezing ability of individual droplets with minimal sample edge effect, we chose a field-of-view (476 μm × 356 μm) at the central region of the sample. Initially, the hierarchical surface stays in a dropwise condensation stage (Fig. 1c), with small spherical condensate droplets growing over time and departing from the surface at an average diameter of ~25 μm. The droplet departure occurs either in the format of out-of-plane jumping or random sweeping^39^,^46^,^47^,^48^,^49^,^50^,^51^,^52^,^53^. Such a dynamic behavior is exemplified by a cluster of condensate droplets circled with green dashed lines as shown in Fig. 1c at 169 s. At 170 s, these droplets disappear as a result of droplet coalescence. Owing to the consistent droplet departure, direct freezing of droplets on the hierarchical surface is rare. The condensate droplets within the field-of-view maintain their liquid state until a freezing wave invading the field-of-view from the sample edge corners at 1410 s (defined as liquid state time *t_l_*), as evidenced by the rapid onset of opacity in droplet 1 circled by the red dashed line. Henceforth, the frozen droplet sprouts dendritic ice crystal (freezing front) towards the surrounding unfrozen liquid droplets, with the ice connection to liquid droplet 2 at 1600 s (Fig. 1d). The freezing of droplet 2 subsequently triggers a new ice crystal as well as an inter-droplet freezing wave that eventually propagates over the entire surface (see the red dashed lines) in a chain reaction fashion. The freezing duration (*t_f_*) from the initial onset of droplet freezing to all droplets freezing within the field-of-view is 395 s. Both two time scales (*t_l_* and *t_f_*) are correlated with the freezing front propagation velocity (V). Although the initial location where the freezing wave intrudes into the field-of-view is impossible to visualize, the average propagation velocity of the freezing wave (V) can be roughly quantified by calculating the ratio between the width of the field-of-view (356 μm) to the freezing duration *t_f_* for the freezing wave to spread over the entire surface. Here, V is ~0.9 μm/s for the hierarchical superhydrophobic surface. As a comparison, we also investigated the droplet freezing and inter-droplet freezing wave propagation on the nanograssed superhydrophobic surface with contact angle of ~160° and contact angle hysteresis of 1 ~ 2°. Condensate liquid droplets on the nanograssed surface exhibit remarkable jumping (Fig. 1e), though relatively less frequent compared to those on the hierarchical surface. We did not observe the droplet freezing in the field-of-view until at *t_l_* ≈ 1090 s. After that, the inter-droplet freezing wave propagates over the entire field-of-view within ~260 s (*t_f_*) (Fig. 1f), corresponding to a V of ~1.4 μm/s. Such a propagation velocity is ~1.5 times faster than that on the hierarchical surface, indicating the effectiveness of the hierarchical roughness on the suppression of inter-droplet freezing wave propagation.

**Figure 2:**
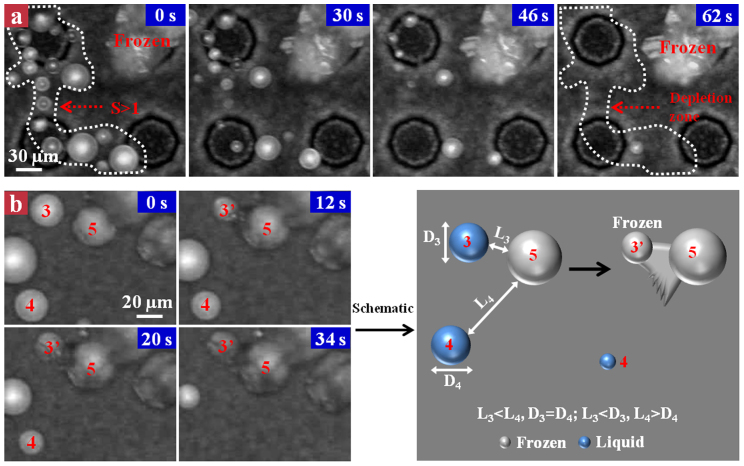
Microscopic visualization of liquid droplet evaporation and ice bridging dynamics. (a) Selected snapshots showing the detailed droplet evaporation and ice bridging dynamics on the hierarchical superhydrophobic surface. All the liquid droplets circled with white dashed line with diameter ranging from ~15 to ~37 μm evaporate (from 0 s to 46 s) without successfully bridging to the invading frost, eventually forming a “depletion zone” at ~62 s. (b) Selected snapshots showing the droplet evaporation and ice bridging dynamics on the nanograssed superhydrophobic surface. Droplet 3 with a S < 1 is successfully frozen by the droplet 5 at the time point of 12 s, whereas droplet 4 with a S > 1 gradually disappears within 34 s without a connection with the droplet 5. (c) Schematic drawing illustrating the dependence of droplet evaporation and ice bridging on the bridging parameter.

Next, we examined the microscopic ice nucleation and freezing wave propagation dynamics on the hierarchical superhydrophobic surface. Figure 2a shows the co-existence of a frozen droplet (ice) and several small liquid droplets with diameters ranging from ~15 to ~37 μm in the field-of-view (see the white dashed circle). During the droplet freezing process, liquid droplets gradually decrease their size in the form of evaporation (from 0 s to 62 s) without successfully bridging to the invading frozen ice. The evaporation of liquid droplets further increases the separation between the invading freezing front and liquid droplet, and as a result, all the liquid droplets completely evaporate without the connection to the freezing front, forming a “depletion zone”^28^,^29^. The preference of liquid evaporation over ice bridging might be explained by considering the interplay between the evaporative liquid droplet and frozen ice. Since the saturation vapor pressure over the frozen ice is much lower than that over the liquid droplet, a vapor gradient from the liquid droplet to the frozen ice is developed. Accordingly, the frozen ice serves as a sink to efficiently adsorb the evaporative water vapor diffused from the vapor source (liquid droplet) in the form of growing ice crystal towards the vapor source direction. From the diffusion point of view, the growth rate of ice crystal is approximately equal to the evaporation rate of the liquid droplet. Thus, the success of ice bridging between the liquid source and frozen sink might involve a length competition between the liquid droplet diameter D and frozen ice-to-liquid droplet separation L (straight-line distance). To quantify the influence of D and L on the feasibility of ice bridging, we defined S = L/D as a bridging parameter. Experimentally, we found that the values of the bridging parameter corresponding to all the liquid droplets within the field-of-view are much larger than unity, suggesting that a smaller L relative to D might be an important signature for a successful ice bridging^28^. This observation is further confirmed by the droplet freezing experiment on the nanograssed superhydrophobic surface. Figure 2b shows a frozen droplet (droplet 5) propagating towards two liquid droplets (droplets 3 and 4) of the same size (D_3_ = D_4_ ≈ 28 μm) but different separation (L_3_ ≈ 9 μm, L_4_ ≈ 53 μm). The droplet 3 is corresponding to S < 1, whereas the droplet 4 is associated with S > 1. Indeed, we found that droplet 3 was frozen at 12 s while droplet 4 completely disappeared within 34 s without connecting to the ice crystal of droplet 5. The schematic drawing in Fig. 2c clearly illustrates the dependence of droplet evaporation and ice bridging on the bridging parameter as reflected in Fig. 2b. Thus, the success of ice bridging between the frozen and unfrozen droplets is sensitive to the length competition between the liquid droplet size (D) and its separation from the nearest frozen sink (L), with a S < 1 favorable for ice bridging.

**Figure 3:**
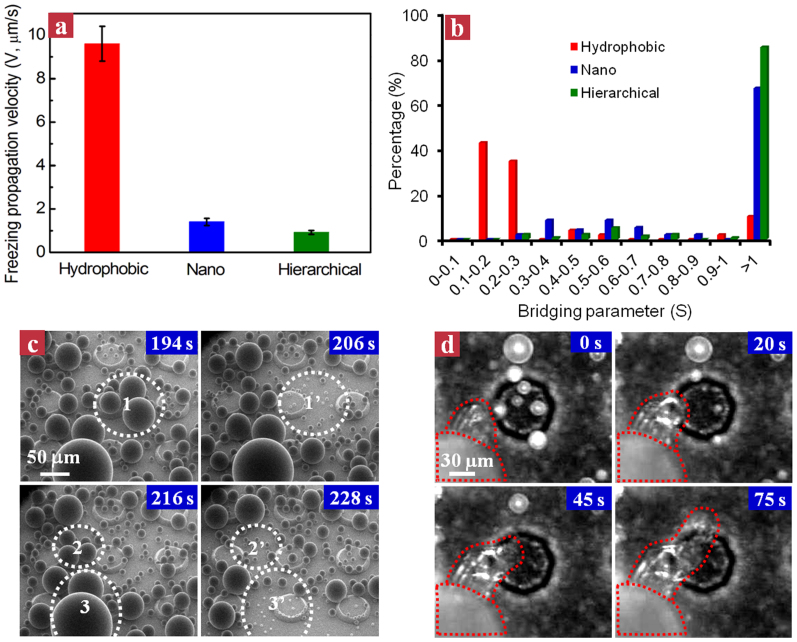
The effects of micro/nanoscale roughness on the suppression of inter-droplet freezing wave propagation. (a) Diagram showing the inter-droplet freezing wave propagation velocities on the hydrophobic, nanograssed and hierarchical surfaces. (b) Histogram of the bridging parameter S on three surfaces during the freezing front propagation process. The hierarchical surface exhibits the largest percentage of droplets with S > 1 (~85%), revealing that the majority of droplets completely evaporate rather than forming an ice bridging with the invading freezing front. (c) Time-lapse ESEM images of condensation on the hierarchical surface. We observed that more than 90% of the droplet departure takes place on the convex corners (see the white dashed circles). (d) Selected snapshots showing the inter-droplet freezing front propagation on a single micro-truncated cone. In order to freeze neighboring liquid droplets that are localized on or behind an inclined cone, the freezing front has to trespass an additional geometric barrier, extending the actual propagation distance (L).

To further correlate the individual droplet behaviors on different surfaces with their macroscopic inter-droplet freezing wave propagation velocities (Fig. 3a), we measured the diameters of all the liquid droplets within the field-of-view before their onset of evaporation as well as their separation from the nearest frozen droplet during the freezing wave spreading process. Figure 3b plots the histogram of bridging parameter S = L/D for all the liquid droplets during the whole freezing process within the field-of-view. It is obvious that ~85% of liquid droplets on the hierarchical superhydrophobic surface are corresponding to S > 1 as opposed to ~67% on the nanograssed surface. Moreover, we found that the majority of evaporative droplets with S > 1 on the hierarchical surface are smaller than 20 μm in diameter. This may be due to the fact that a small droplet corresponds to a higher vapor saturation pressure according to the Kelvin equation, thus during the freezing process, the small droplet tends to evaporate completely without a successful connection to the surrounding frozen droplet.

### Suppression of inter-droplet freezing wave propagation using microscale edge effect

The large percentage of droplets with S > 1 on the hierarchical superhydrophobic surface might be endowed by the incorporation of well-controlled microscale structures on the hierarchical surface, which provides adequate active nucleation sites to reinforce the droplet departure. Without controlled nucleation sites, droplet departure occurs in a random fashion. On a flat hydrophobic surface (Supplementary Fig. S1), we did not observe any spontaneous droplet departure and the measured inter-droplet freezing propagation velocity is ~9.4 μm/s, which is one order of magnitude faster than that on the two-tier surface. Although we observed pronounced droplet jumping on the one-tier surface, its departure frequency is much lower relative to that on the hierarchical surface. In contrast, owing to the increased coalescence engendered by the spatial control of heterogonous nucleation sites at the convex edges^39^,^44^,^54^,^55^, droplet departure is substantially accelerated. Indeed, based on the environmental scanning electron microscopy (ESEM) visualization (Fig. 3c), we observed that more than 90% of the droplet departure takes place on the convex corners (see the white dashed circles). Owing to such an enhanced departure dynamics on the hierarchical surface, the average liquid droplet size D before the onset of evaporation is decreased whereas the separation between the liquid droplet and nearest frozen droplet L is increased. For example, during the freezing wave spreading process, the average D and L on the nanograssed surface are ~23 μm and ~33 μm, respectively, corresponding to a value of S ≈ 1.4 > 1. In contrast, the S on the hierarchical surface is ~3.0 (D ≈ 16 μm and L ≈ 49 μm), suggesting the influence of enhanced droplet departure manifested on the hierarchical surface on the bridging parameter distribution.

Moreover, the incorporation of the three-dimensional (3-D) microscale structures with inclined edges on the hierarchical superhydrophobic surface also limits the success of ice bridging. Figure 3d shows the inter-droplet freezing wave propagation on a single micro-truncated cone. To freeze droplets located on or behind inclined micro-truncated cones, the freezing front has to overcome an additional geometric barrier imposed by the inclined micro-truncated cone, as revealed by the ice bridging in the upward (0 ~ 20 s) and downward (45 ~ 75 s) directions. Thus, unlike the freezing wave propagation on the surface with nanoscale roughness alone, the actual inter-droplet freezing wave propagation pathway or liquid droplet-to-frozen droplet separation (L) is accordingly elongated. Taken together, the favorable bridging parameter distribution and enlarged L endowed by the controlled edge effect permit more individual liquid evaporation than ice bridging, thereby leading to a collective yet disordered inter-droplet freezing wave propagation and significantly suppressing the freezing wave propagation at the whole surface level.

### Defrosting results

**Figure 4:**
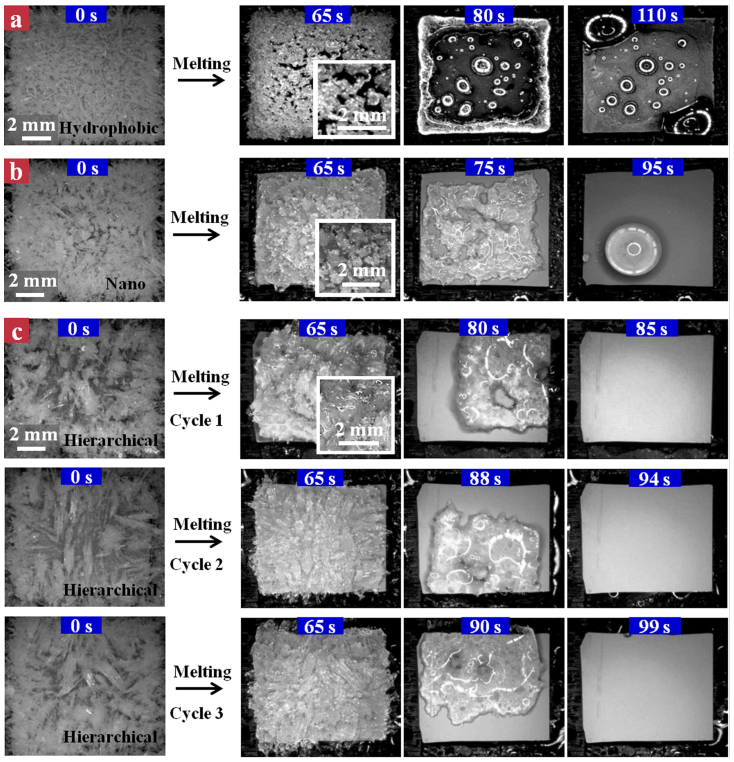
Selected snapshots showing the frost morphology evolution over time on three different substrates. (a) Flat hydrophobic surface: After one hour of condensation frosting experiment, the whole hydrophobic surface is covered by thick frost layer (time: 0 s). The frost is fractured into many irregular pieces during the defrosting stage, which can be clearly seen in zoom-in images at 65 s. With the time progression, the fractures avalanche in a domino-like process, leading to the formation of enlarged cracks, as shown in the image at 80 s. Finally, the hydrophobic surface is covered by many scattered sticky water droplets (~110 s). (b) Nanograssed superhydrophobic surface: During the defrosting process, the nanograssed surface exhibits a relative low fracture density and the melting frost becomes a large spherical water droplet at the culmination of defrosting (~95 s). (c) Hierarchical superhydrophobic surface: During the defrosting stage, the frost preserves its integrity without the formation of visible cracks and eventually detaches from the surface within a relatively shorter time (~85 s) in the first frosting/defrosting cycle. The unique defrosting behavior is sustained in the repetitions of frosting/defrosting experiments (see the defrosting cycles 2 and 3). Moreover, there is no obvious degradation in the defrosting time, suggesting the remarkable stability of the hierarchical surface in sustaining its efficient defrosting behavior.

Despite promising prospect in the retardation of frost formation demonstrated on our hierarchical micro/nano-engineered surface, the frost formation is inevitable over long duration of freezing. Indeed, after an hour of freezing on the −10°C cooling stage, we observed that all the cooled surfaces were covered by thick porous frost (see the time point of 0 s in Fig. 4a–c). Yet it is apparent that the frost on the hierarchical surface (Fig. 4c) appears much spongy compared to those on the hydrophobic and nanograssed surfaces, owing to the delayed frost formation and growth. To remove the frost from these samples, we raised the temperature of the cooling stage to 0°C. On the hydrophobic surface (Fig. 4a), we observed that the bulk frost is fractured into many irregular pieces (see the zoom-in images at 65 s) upon melting. With the time progression, the fractures avalanche in a domino-like fashion, leading to the formation of enlarged cracks and hence a decreasing fracture density, as shown in the image of Fig. 4a at 80 s. Finally, the hydrophobic surface is covered by many scattered sticky water droplets (~110 s). In contrast, during the defrosting process, the nanograssed surface exhibits a relative low fracture density and the melting frost becomes a large spherical water droplet at the culmination of defrosting (~95 s, Fig. 4b), similar to the results observed by Boreyko *et al*^56^. These retained droplets on the hydrophobic and nanograssed surfaces serve as active freezing sites in the subsequent frosting process, which not only facilitate the unwanted frosting process, but also lead to a significant increase in the defrosting time (Supplementary Fig. S2 and Fig. S3). For example, in the case of the hydrophobic surface, the defrosting time in the third frosting/defrosting cycle is ~360 s, which is over 3 times longer than that in the first defrosting process (~110 s). Such a considerable degradation in its defrosting ability poses severe challenges for practical applications^57^,^58^,^59^,^60^. Interestingly, on the hierarchical surface we didn't observe any visible fractures in the defrosting stage (Fig. 4c). The frost layer detaches from the surface within a relatively shorter time (~85 s) preserving its initial bulk shape, suggesting there may exist a lubricating layer between the frost and the underlying solid interface. The unique defrosting pattern is maintained in more repetitions of defrosting experiments (three cycles for demonstration). Moreover, there is no obvious degradation in the defrosting time (cycles 1 ~ 3 in Fig. 4c), suggesting the remarkable stability of the hierarchical surface in sustaining the efficient defrosting behavior.

## Discussion

Previously we discussed the influence of micro/nanoscale roughness on the delay of frost formation and inter-droplet freezing wave propagation. In particular, we demonstrated that the controlled-introduction of microscale structures with inclined edges not only reinforces spontaneous condensate droplet departure behavior for delaying frost growth, but also increases additional structural barrier for the ice bridging, both of which contribute to the suppression of inter-droplet freezing wave propagation. Note that these spatially controlled micro-edges are distinctively different from those large sample edges defining the geometry boundary of our samples which are associated with low heterogeneous ice nucleation energy barrier owing to their geometric singularity. In contrast, the patterned micro-edges dramatically impede individual frost formation and inter-droplet freezing wave propagation through a continuous process of droplet nucleation, coalescence, departure, and/or evaporation.

**Figure 5:**
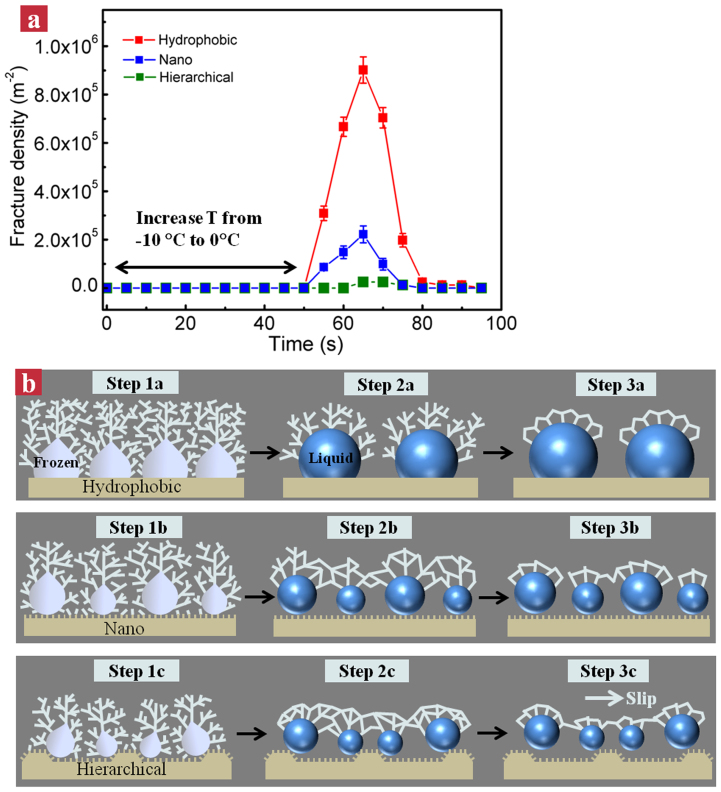
The effects of surface roughness on the frost removal. (a) The time evolution of fracture densities on three different surfaces. (b) Schematic drawing showing the frost morphology evolution on three different substrates during the defrosting process. Owing to the remarkable large adhesion between the melting frost and the underlying substrate, random fractures emerge in the frost on the flat hydrophobic surface (step 1a–3a). In contrast, the incorporation of nanoscale roughness in the nanograssed or hierarchical surfaces increases the lubrication of liquid between the frost and underlying solid interface (step 1b–3b, 1c–3c), preventing the surfaces from severe fractures. Moreover, the presence of 3-D, inclined microstructures on the hierarchical surface (step 1c–3c) further facilitates the frost movement, promoting the frost removal with an enhanced structural integrity (step 3c).

To explain the efficient frost removal on the hierarchical surface, we quantified the variation of fracture density over time. The fracture density is defined as the number of visible fractures per unit area during the defrosting process. Among three samples studied, the flat hydrophobic surface displays the maximum fracture density during the defrosting process (Fig. 5a). Owing to the small contact angle (~110°), the flat hydrophobic surface yields a strong affinity to the frozen ice and melting liquid, and accordingly the frozen ice and melting liquid are tightly anchored at the bottom surface with a strong adhesion (step 1a–3a in Fig. 5b). In contrast, the frost at the upper layers has a relatively weak interaction. Thus, it is expected that the large difference in interaction at the bottom and upper layers leads to the emergence of pronounced fractures upon defrosting. In contrast, the incorporation of nanosacale roughness in the nanograssed or hierarchical surfaces contributes to the formation of “Cassie-droplet” during the defrosting process (step 1b–3b, 1c–3c in Fig. 5b), resulting in the formation of interconnected network without encountering the severe fracture as opposed to that on the hydrophobic surface. Notably, the incorporation of 3-D microscale edge structures aids the mobility of the melting underneath bulk frost layer in both lateral and vertical directions (step 1c–2c in Fig. 5b). Moreover, coupled with the minimal frost adhesion to the substrate enabled by the trapped air pockets in the nanoscale roughness, the enhanced mobilization allows for the preservation of the integrity of the whole frost layer during the defrosting process. As a result, the melting droplets at the micro/nanostructured interface serve as liquid lubricant, accelerating the efficient removal of frost layer as described above (step 3c in Fig. 5b). Whereas without the 3-D structure and two-tier roughness, the melting water droplets on the nanograssed surface retain with spherical shapes, and don't slip away from the surface (step 3b in Fig. 5b). We expect the efficient frost removal on the hierarchical surface endowed by the synergic cooperation of micro/nano-scale roughness and the microscale edge effect could dramatically lower the energy cost associated with the defrosting.

In summary, we developed a robust icephobic surface that allows for enhanced frost formation retardation through the suppression of inter-droplet freezing propagation over the entire surface, as well as efficient frost removal by self-lubrication. Our work investigates both frosting and defrosting processes on engineered icephobic surfaces in an integrated approach. We demonstrate that these improved performances are ascribed to the microscale edge effect and the synergistic cooperation of micro/nano-scale roughness. In particular, we find that the spatial control of microscale edges in the hierarchical surface not only increases the energy and structural barriers for the interdroplet freezing wave propagation through a continuous process of droplet nucleation, coalescence, departure and/or evaporation, but also enhances the bulk frost mobility during the defrosting stage. We envision that the concept of harnessing surface morphology to achieve superior performances in two opposite phase transition processes (frosting/defrosting) might open up a new avenue for the development of efficient materials for various applications ranging from anti-icing, dropwise condensation and water harvesting.

## Methods

### Fabrication of hierarchical superhydrophobic surface

The hierarchical surface with nanograssed micro-truncated cone architecture was fabricated using a combined anisotropic wet-etching and deep reactive ion etching (DRIE) process. First, a pattern defining the microscale feature was first created in silicon wafer using standard photolithography and then wet etched in 25 wt.% Tetramethylammonium hydroxide (TMAH) solution at a working temperature of 80°C, achieving a surface with the micro-truncated cone architecture with an inclination angle of 54.7°. Next, a modified Bosch DRIE process was used to fabricate uniform nanograsses on the whole surface. This dry etching process is based on a sequential passivation and etching cycle. By alternating the etching and passivation steps, nanograss arrays with the diameter of ~300 nm, height of ~5 μm, and pitch of ~200–350 nm were formed. The as-fabricated sample was then immersed in 1 mM hexane solution of perfluorooctyl trichlorosilane for ~30 min, followed by heat treatment at ~150°C in air for one hour to tailor its surface superhydrophobic. For comparison purposes, the flat hydrophobic surface and superhydrophobic surface with nanograss alone were also fabricated.

### Condensation frosting experiment

The condensation frosting experiment was carried out in an environment with the ambient temperature of 22 °C and relative humidity of 65%. Initially, the samples of 9 mm × 9 mm were horizontally placed onto a cooling stage with a preset temperature of −10 °C. We visualized the frost formation using a digital microscope (Keyence/VHX-1000) equipped with a universal zoom lens VH-Z100UR/Z100UW. To observe the droplet freezing dynamics with the minimal edge effect, we focused the microscope lens at the central region of the sample with a field-of-view of 476 μm × 356 μm. The droplet condensation and subsequent freezing process were recorded by the CCD camera at a frame rate of 15 fps.

### Defrosting process

After one hour condensation frosting on the −10°C cooling stage, we raised the temperature of the cooling stage to 0 °C at ~15 °C/min. We observed the defrosting dynamics on the entire surfaces using a ultra-small, high-performance zoom lens VH-Z20R/Z20W, corresponding to a field-of-view of 18 mm × 14 mm. When the frosts on the surfaces were completely melted to liquid water (in the case of flat hydrophobic surface and nanograssed surface) or detached (in the case of hierarchical surface), we reduced the temperature of the cooling stage back to −10 °C at 1.2 °C/min to allow for the re-initiation of a new frosting process. Totally three cycles of frosting/defrosting process were conducted to test the defrosting ability of the surfaces.

### Observation of dropwise condensation dynamics using ESEM

The droplet departure dynamics on the hierarchical superhydrophobic surface was in situ visualized using a Philips XL-30 ESEM. The water vapor pressure in the ESEM chamber was set at ~5.1 Torr, and the temperature of the peltier cooling stage was fixed at ~1°C. To minimize beam heating effect, we chose the electron beam voltage of 15 keV and the view areas were ~307 μm × 410 μm.

## Author Contributions

Z.W. and X.C. conceived the concepts of the research. X.C., R.M., H.Z., X.Z. and L.C. prepared the samples. X.C. designed and performed the experiments, collected and analyzed the data. Z.W., X.C. and S.Y. were responsible for writing the paper. All authors contributed to the manuscript.

## Supplementary Material

Supplementary Information Supplementary Information
