# Supplementary material for: Activating the Microscale Edge Effect in a Hierarchical Surface for Frosting Suppression and Defrosting Promotion
Source: Sci Rep. 2013 Aug 28;3:2515. doi: 10.1038/srep02515 (PMC3755279; doi:10.1038/srep02515)
Supplement: Supplementary Information [file srep02515-s1.pdf]

Supplementary Information

Activating the Microscale Edge Effect in a Hierarchical Surface for Frosting Suppression and Defrosting Promotion

Xuemei Chen,[1] Ruiyuan Ma,[2] Hongbo Zhou,[2] Xiaofeng Zhou,[3] Lufeng Che,[3] Shuhuai Yao,[2]* & Zuankai Wang[1,4]*

1 Department of Mechanical and Biomedical Engineering, City University of Hong Kong, Hong Kong 999077, China

2 Department of Mechanical Engineering, The Hong Kong University of Science and Technology, Hong Kong 999077, China

3 State Key Laboratory of Transducer Technology, Shanghai Institute of Microsystem and Information Technology, Chinese Academy of Sciences, 865 Changning Road, Shanghai 200050, People's Republic of China

4 Shenzhen Research Institute of City University of Hong Kong, Shenzhen, China

Correspondence and requests for materials should be addressed to:
S. Y. (meshyao@ust.hk) or Z. W. (zuanwang@cityu.edu.hk)

Condensation Frosting Experiment on the Hydrophobic Surface

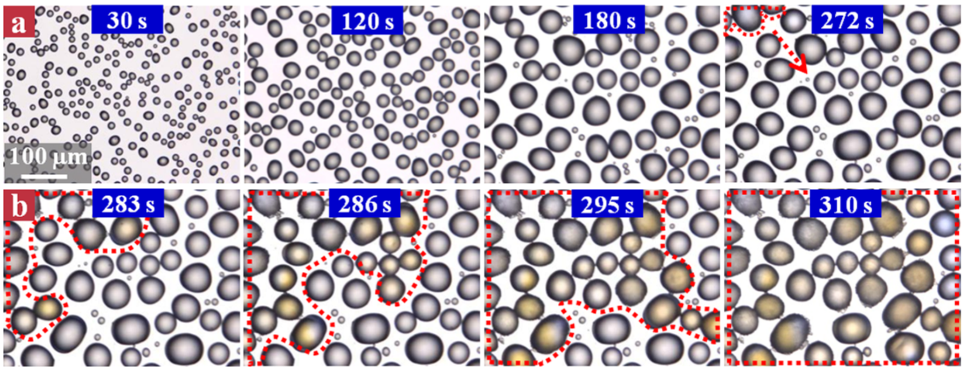

Figure S1 │ Condensation frosting process on the hydrophobic surface. (a) Droplet freezing dynamics on the hydrophobic surface chilled at -10°C. The condensate droplets grow faster, and don't depart from the surface. Condensate droplets maintain their liquid state until a freezing wave invading the field-of-view at ~272 s. (b) Selected snapshots showing the inter-droplet freezing wave propagation across the field-of-view. The inter-droplet freezing wave gradually propagates across the entire field-of-view within ~ 38 s, which are ~7 and ~10 times faster than those on the nanograssed and hierarchical surface, respectively.

As a comparison, we also investigated the condensation frosting dynamics on the flat hydrophobic surface. We found that the droplet freezing and freezing wave propagation on the hydrophobic surface are distinctly different from those on the nanograssed and hierarchical surfaces. First, on the hydrophobic surface, the time required for the inter-droplet freezing wave to invade the center part of the sample (field-of-view is as same as previously) is ~272 s (Figure S1a). The short freezing time is due to the absence of spontaneous droplet jumping and fast droplet nucleation and growth on the flat surface. The inter-droplet freezing front propagation velocity V is measured to be ~9.4 μm/s (Figure S1b), which is ~7 and ~10 times larger than those on the nanograssed and hierarchical surfaces, respectively. This experiment on the hydrophobic surface reveals the importance of nanoscale roughness in the suppression of inter-droplet freezing wave spreading.

Defrosting Repetitions on the Hydrophobic and Nanograssed Surfaces

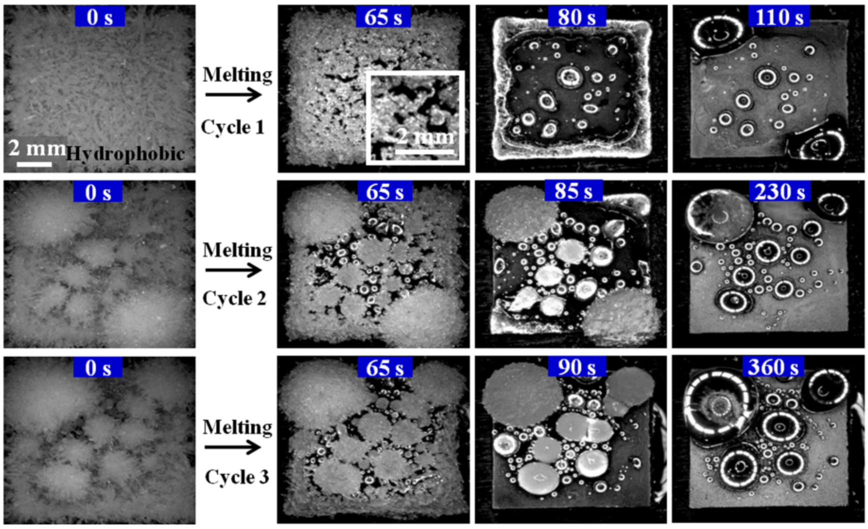

Figure S2 | Selected snapshots showing the frost morphology evolutions over time for 3 repetitions of defrosting experiments on the flat hydrophobic surface. During the first defrosting experiment, the frost is fractured into many irregular pieces (see the zoom-in images at 60 s), with the time progression, the fractures avalanche in a domino-like process, leading to the formation of enlarged cracks, as shown in the image at 80 s. Finally, the hydrophobic surface is covered by many scattered sticky water droplets (~110 s). These retained scattered droplets on the hydrophobic surface are vulnerable to subsequent freezing, which not only facilitate the unwanted frosting process (see the defrosting cycles 2 and 3), but also lead to a significant increase in the defrosting time.

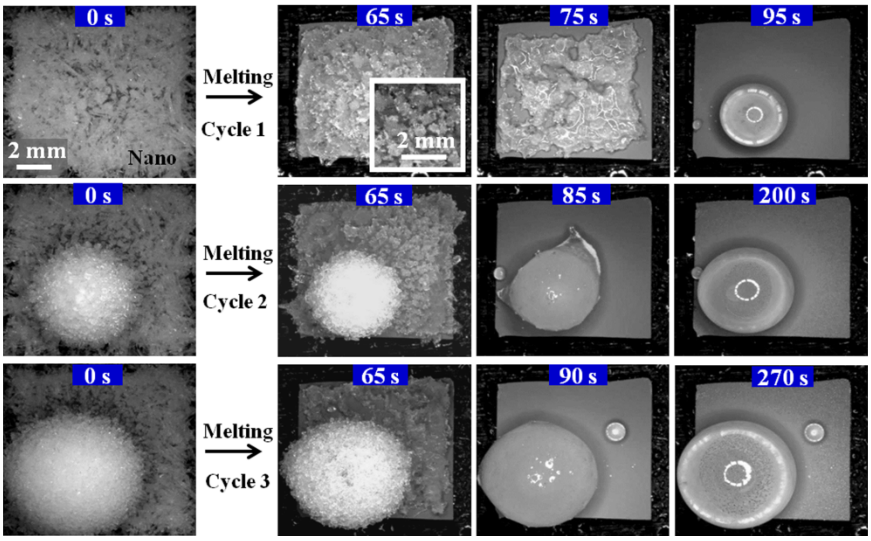

Figure S3 | Selected snapshots showing the frost morphology evolutions over time for 3 repetitions of defrosting experiments on the nanograssed surface. During the first defrosting process, the nanograssed surface exhibits a relative low fracture density and the melting frost becomes a large spherical water droplet at the culmination of defrosting (~ 95 s). The large spherical droplet is vulnerable to subsequent freezing (see the defrosting cycles 2 and 3), which poses challenges for practical application.

Figures S2 and S3 show the selected snapshots of frosting/defrosting process on the flat hydrophobic and nanograssed surfaces, respectively. Without the presence of hierarchical roughness, the melting droplets are prone to sticking to or staying on the surfaces (the surfaces are horizontally placed). When undergoing a re-frosting process under the same frosting condition (we reduced the temperature of the cooling stage back to -10 °C at 1 °C/min), these retained liquid droplets on the hydrophobic and nanograssed surfaces serve as favorable freezing sites, leading to severe freezing in the subsequent re-frosting process (see the defrosting cycles 2 and 3 in Figures S2 and S3). As a result, the defrosting time in the subsequent cycle becomes elongated. For example, on the hydrophobic surface, the time to melt the frost into water droplets in the first defrosting process is ~110 s, which is over 3 times shorter than that in the third defrosting process (~ 360 s), indicating the obvious degradation in its defrosting ability.
